# Culture-Free Detection of Antibiotic Resistance Markers from Native Patient Samples by Hybridization Capture Sequencing

**DOI:** 10.3390/microorganisms9081672

**Published:** 2021-08-05

**Authors:** Ines Ferreira, Sarah Lepuschitz, Stephan Beisken, Giuseppe Fiume, Katharina Mrazek, Bernhard J. H. Frank, Silke Huber, Miriam A. Knoll, Arndt von Haeseler, Arne Materna, Jochen G. Hofstaetter, Andreas E. Posch, Johannes Weinberger

**Affiliations:** 1 Ares Genetics GmbH, 1030 Vienna, Austria; ines.ferreira@ares-genetics.com (I.F.); sarah.lepuschitz@ares-genetics.com (S.L.); stephan.beisken@ares-genetics.com (S.B.); fiume.gius@gmail.com (G.F.); kathi.mrazek@live.at (K.M.); arne.materna@ares-genetics.com (A.M.); andreas.posch@ares-genetics.com (A.E.P.); 2 Center for Integrative Bioinformatics Vienna, Max Perutz Laboratories, University of Vienna, 1030 Vienna, Austria; arndt.von.haeseler@univie.ac.at; 3 Center for Integrative Bioinformatics Vienna, Max Perutz Laboratories, Medical University of Vienna, 1030 Vienna, Austria; 4 Michael Ogon Laboratory for Orthopaedic Research, Orthopaedic Hospital Vienna-Speising, 1130 Vienna, Austria; Bernhard.Frank@oss.at (B.J.H.F.); Jochen.Hofstaetter@oss.at (J.G.H.); 5 Institute of Hygiene and Medical Microbiology, Medical University of Innsbruck, 6020 Innsbruck, Austria; silke.huber@i-med.ac.at (S.H.); miriam.knoll@i-med.ac.at (M.A.K.); 6 Bioinformatics and Computational Biology, Faculty of Computer Science, University of Vienna, 1090 Vienna, Austria

**Keywords:** target enrichment, antimicrobial resistance, next generation sequencing, human pathogens, urinary tract infection, synovial fluid, infectious disease surveillance, molecular detection

## Abstract

The increasing incidence of antimicrobial resistance (AMR) is a major global challenge. Routine techniques for molecular AMR marker detection are largely based on low-plex PCR and detect dozens to hundreds of AMR markers. To allow for comprehensive and sensitive profiling of AMR markers, we developed a capture-based next generation sequencing (NGS) workflow featuring a novel AMR marker panel based on the curated AMR database ARESdb. Our primary objective was to compare the sensitivity of target enrichment-based AMR marker detection to metagenomics sequencing. Therefore, we determined the limit of detection (LOD) in synovial fluid and urine samples across four key pathogens. We further demonstrated proof-of-concept for AMR marker profiling from septic samples using a selection of urine samples with confirmed monoinfection. The results showed that the capture-based workflow is more sensitive and requires lower sequencing depth compared with metagenomics sequencing, allowing for comprehensive AMR marker detection with an LOD of 1000 CFU/mL. Combining the ARESdb AMR panel with 16S rRNA gene sequencing allowed for the culture-free detection of bacterial taxa and AMR markers directly from septic patient samples at an average sensitivity of 99%. Summarizing, the newly developed ARESdb AMR panel may serve as a valuable tool for comprehensive and sensitive AMR marker detection.

## 1. Introduction

The burden of antimicrobial resistance (AMR) and its accelerating progression has been acknowledged worldwide by leading health institutes such as the WHO and the CDC [1,2,3]. Besides the need for new antibiotics, efforts in the field of AMR surveillance and diagnostics are essential to address the rise of AMR [4].

One unmet need to inform AMR surveillance is to comprehensively define resistomes in emerging multidrug-resistant human pathogens [5]. The WHO report on AMR surveillance [1] states that next generation sequencing (NGS) of bacterial isolates serves as a valuable tool for molecular AMR profiling. In order to obtain a more comprehensive picture of the prevalence, transmission, and composition of AMR, it would be beneficial to extend AMR profiling beyond bacterial culture and enable AMR profiling directly from native samples. Thereby, monitoring gaps caused by non-culturable bacteria or false negative culture results could be reduced. However, culture-free AMR profiling methods need to overcome certain challenges [6]. For example, the fraction of host DNA in clinical specimens outweighs microbial DNA by nine orders of magnitude. Ultra-deep sequencing has been proposed as a solution to address this issue, but excessive sequencing costs and an uncertain performance when screening for individual AMR markers in low abundance microbes hamper routine use [7]. Other culture-free technologies such as PCR-based or loop mediated isothermal amplification (LAMP)-based assays have been demonstrated to sensitively detect AMR markers; however, their target space usually comprises only a limited set of markers for only a few dozen AMR markers [8].

**Figure 1:**
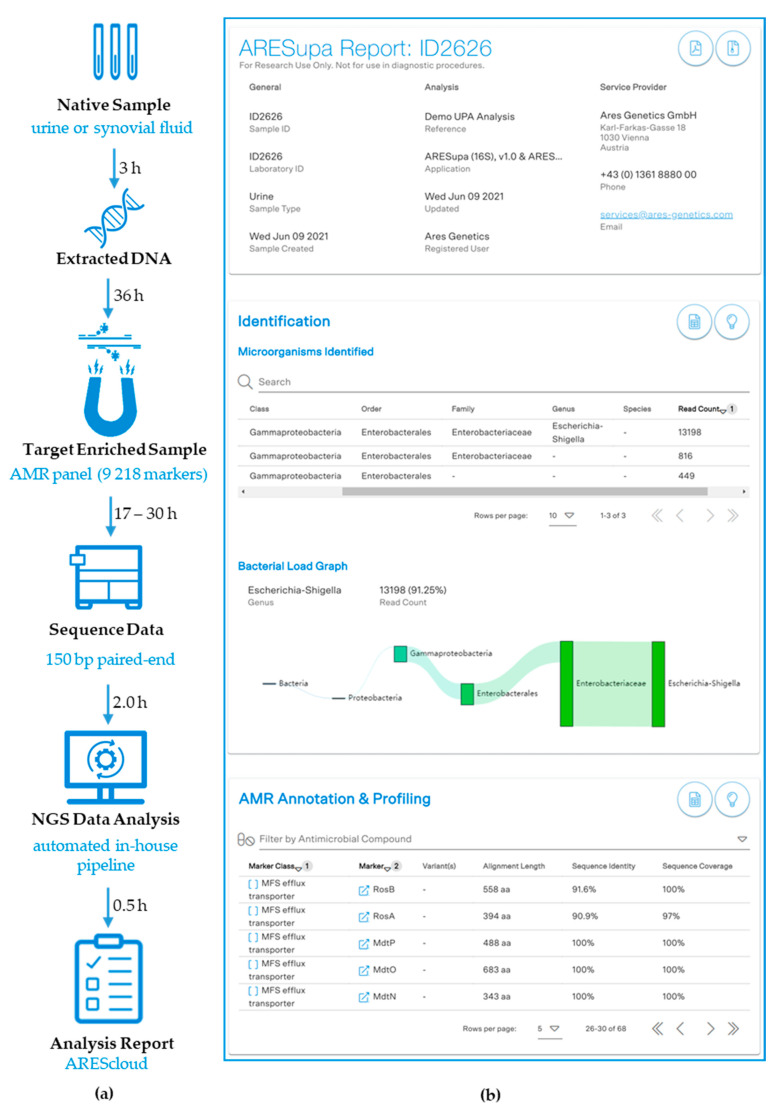
Workflow from sample to result. (**a**) Flow chart of the target enrichment workflow established and tested for synovial fluid and urine samples. (**b**) Example of the standardized sample report implemented on AREScloud to summarize AMR marker profiling results and taxonomic identification via 16S rRNA sequencing.

To address these constraints for culture-free AMR marker detection, we established a capture-based next generation sequencing (NGS) workflow selectively enriching 9218 AMR markers catalogued in ARESdb for samples with low bacterial biomass or a high background of host DNA. In order to assess the sensitivity of the ARESdb AMR panel, we determined the limit of detection (LOD) by spike-in experiments in different native specimens, and subsequently performed an initial proof-of-concept in a set of septic patient samples. In addition, the workflow was complemented with 16S rRNA gene sequencing to enable taxonomic profiling (Figure 1).

## 2. Materials and Methods

### 2.1. Clinical Samples and Bacterial Isolates

#### 2.1.1. Aseptic Urine and Synovial Fluid Samples

Aseptic urine samples were collected from three healthy male and three healthy female probands. To investigate the differences among female and male samples, one urine pool sample was created from male probands and one urine pool sample from female probands. Further, aseptic synovial fluids from six patients who underwent revision arthroplasty after total knee joint replacement were included. Synovial samples were retrieved under sterile conditions by aspiration of the affected joint in the operating theater. Samples were collected between 09/2019 and 07/2020 at the Orthopaedic Hospital Vienna-Speising. Synovial fluids were considered as aseptic according to the following criteria: aseptic indication for revision arthroplasty, no clinical signs of infection, negative laboratory infection parameters, negative aerobic and anaerobic microbiological cultures and fungi cultures, and aseptic histological results. After joint aspiration, six aseptic synovial fluid samples were stored at −80 °C and shipped frozen on dry ice to the testing laboratory for further analysis. As volumes of synovial fluid were limited (five to eight milliliters per patient) and in order to obtain sufficient sample material for all planned spike-in experiments, synovial fluids were pooled from all available patient samples.

To determine the LOD, reference strains of two Gram-negative (*E. coli* ATCC 35218, *K. quasipneumoniae* ATCC 700603) and two Gram-positive pathogens (*S. aureus* ATCC BAA-2312, *E. faecium* ATCC 700221) were used for spike-in experiments. All samples were processed as duplicates and native blank control samples with no bacterial spike-in were included. Bacteria present in unspiked aseptic sample material, i.e., controls, were considered background and removed for the analysis of the LOD. Values for performance metrics were averaged across replicates.

#### 2.1.2. Septic Urine Samples

Septic urine samples were residue samples collected from routinely processed urine samples derived from patients with suspected UTI (urinary tract infection) at the Institute of Hygiene and Medical Microbiology at the Medical University of Innsbruck. Isolates from patients with clinical signs of UTI were primarily selected based on macroscopic turbidity of urine samples as well as microscopic identification of bacterial structures and leukocytes in native samples. Samples were frozen immediately after plating of cultures to ensure freshness and stored before further selection. Isolates of the causative pathogen cultured on selective agar plates were frozen in skim milk. Antimicrobial inhibitor tests were performed for each sample by inoculating antibiotic-free filter plates with 10 µL of native urine and placing them on an agar plate seeded with Bacillus subtilis spores (Axon Lab AG, Polling, Austria). Bacterial cultures were obtained using BD CHROMagar Orientation Medium (Becton Dickinson Diagnostics, Heidelberg, Germany). Colony-forming units (CFUs) counts were read after overnight culture and were classified into categories of below 10,000 CFU/mL, approximately 10,000 CFU/mL, and more than 10,000 CFU/mL. Species identification of relevant pathogens was performed by matrix-assisted laser desorption/ionization time of flight mass spectrometry (MALDI-TOF MS, Bruker Daltonik, Bremen, Germany) using the reference Biotyper library v4.1 (Bruker Daltonik, Bremen, Germany). Samples were included if clinically relevant monocultures were obtained. Thirteen septic urine samples as well as 13 corresponding bacterial isolates were shipped to the testing laboratory (Table 1). All samples were processed as duplicates and values for performance metrics were averaged across replicates.

## 2.2. Wet-Lab Workflow

### 2.2.1. Cultivation, DNA Isolation, and Quantification

A stock solution for each reference strain (*E. coli* ATCC 35218, *K. quasipneumoniae* ATCC 700603, *S. aureus* ATCC BAA-2312, *E. faecium* ATCC 700221) was prepared by picking one colony from the overnight culture and resuspended in 1 mL PBS. Serial dilutions from 10^1^ to 10^7^ were made and an aliquot of 100 µL was subsequently cultured overnight on plate-count agar. The calculation of colony forming units per ml (CFUs/mL) was averaged across triplicates and spike-in dilutions were prepared for 10, 100, 1000, 10,000, and 100,000 CFU/mL. Then, defined concentrations were spiked into aseptic native material (synovial fluid, male-, female-urine) and processed as duplicates.

Automated DNA extractions from native samples and bacterial isolates were performed on a QIAsymphony SP instrument (QIAGEN, Hilden, Germany) using the QIAsymphony DSP DNA Kit (QIAGEN) as described previously [9]. Each independent DNA extraction contained no template controls (NTC) containing molecular grade water only.

Quantitative PCR (qPCR) to assess bacterial and human DNA concentrations was performed with the CFX96 Touch Real-Time PCR System (Bio-Rad, Hercules, CA, USA). The qPCR reaction was performed in a total volume of 20 µL using the TaqMan Universal PCR Master Mix (Applied Biosystems, Bedford, MA, USA), containing 100 nM of each of the forward and reverse primers and the fluorogenic probe. The reaction conditions for amplification of DNA were 50 °C for 2 min, 95 °C for 10 min, and 40 cycles of 95 °C for 15 s and 60 °C for 1 min. Data analysis made use of Bio-Rad CFX Maestro Software. *S. aureus* and *H. sapiens* DNA were used as standards for determining concentrations of samples by real-time PCR. For bacterial detection [10], the reaction set included forward primer (5′-TCCTACGGGAGGCAGCAGT-3′), reverse primer (5′-GGACTACCAGGGTATCTAATCCTGTT-3′), and the probe ((6-FAM)-5′-CGTATTACCGCGGCTGCTGGCAC-3′-(TAMRA)), and for human detection [11], the reaction set included forward primer (5′-CATGGTGAAACCCCGTCTCTA-3′), reverse primer (5′-GCCTCAGCCTCCCCGAGTAG-3′), and the probe ((6-FAM)-5′-ATTAGCCGGGCGTGGTGGCG-3′-(TAMRA)).

### 2.2.2. ARESdb AMR Panel Design

A total of 9218 markers, including 7312 AMR genes and 1906 genetic variants, were extracted from ARESdb (date: 24 June 2020) [12]. Probes (120 bp length) were proprietary designed by Roche (Pleasanton, CA, USA) to cover all genes with a tilling of one, i.e., a targeted per-base coverage of one. Because of an insufficient marker length, five AMR markers were excluded from the panel design (minimum required marker length: 120 bp). The final panel covered a capture space of 7.74 Mbp.

### 2.2.3. Library Preparation and Target Enrichment

Illumina Library preparation of DNA extracts from native samples was performed using the KAPA HyperCap Workflow v3.0 (Roche, Pleasanton, CA, USA). Briefly, 100 ng or up to 35 µL of low-concentrated DNA sample were used for library preparation. Libraries were prepared for sequencing with and without hybridization enrichment using two different sequencing lanes. The manufacturer’s protocol was followed with the following modifications: (1) pre-capture PCR ranged between 13 and 17 cycles based on bacterial DNA concentrations; (2) we applied a hybridization time of 18 h; (3) pooling for hybridization was done based on CFU/mL spike in concentrations (e.g., 8-plex pool of eight 10 CFU/mL spiked in samples); and (4) post-capture PCR ranged between 17 and 20 cycles based on pre-capture PCR concentrations.

Bacterial isolate libraries were prepared for whole genome sequencing using QIAseq FX DNA Library Kit (QIAGEN, Hilden, Germany), as described previously [9].

Each independent library preparation contained a no template control (NTC) containing molecular grade water only. Paired-end sequencing was performed on an Illumina NextSeq550 instrument (Illumina, San Diego, CA, USA) using NextSeq 300-cycle Mid Output Kit v2.5 (Illumina).

### 2.2.4. 16S rRNA Amplicon Sequencing

16S amplicon library prep was performed using the QIAseq 16S/ITS Screening Panel kit (QIAGEN, Hilden, Germany) according to the manufacturer’s protocol. Paired-end sequencing was performed on an Illumina MiSeq instrument (Illumina) using MiSeq 600-cycle Reagent Kit v3 (Illumina).

### 2.3. Dry-Lab Workflow

#### 2.3.1. 16S rRNA Data Analysis

Primer sequences were trimmed using cutadapt v2.6 [13], followed by read QC. Reads below 200 bp with a Phred score below 10 were removed. The DADA2 (1.18.0) [14] pipeline was used to construct amplicon sequence variant (ASV) tables with “filterAndTrim” parameters set to “truncLen = c(220, 180), maxN = 20, maxEE = c(3, 7)”; DADA2 defaults were used for all other parameters. Taxonomic classification was determined using the SILVA [15] rRNA reference database v138 via “assignTaxonomy” followed by “addSpecies”, assigning species information based on exact sequence matching. For reporting, ASV counts were summarized per genus and hits with less than 1% abundance were considered background and removed.

#### 2.3.2. Bioinformatics NGS Data Pipeline

The same NGS pipeline was used for the analysis of enriched as well as metagenomics sequenced samples. Read quality was evaluated with FastQC v0.11.9 and MultiQc v1.10.1 [16,17]; reads were trimmed and quality filtered using Trimmomatic v0.39 with parameters “LEADING:10 TRAILING:10 SLIDINGWINDOW:4:15 MINLEN:36” [18], followed by removal of duplicate reads using FastUniq v1.1 [19]. Meta-assembly of reads was performed using SAUTE v 1.3.0, included in the SKESA assembler package [20]. AMR marker detection was performed on assembled contigs using protein BLAST ncbi-blast v2.9.0+ with parameter “qcov_hsp_perc 60” and subsequent filtering of the blast hits to a minimal identity of 90% [21]. If several AMR proteins aligned to the same area on a contig, the marker with the best bitscore was selected. Because the aseptic material was found to be not sterile, i.e., the aseptic material contained AMR markers also present in strains used as spike-in, markers detected on negative controls and blanks of aseptic material were excluded from the comparison to avoid inflating sensitivity. The marker sets considering background were separately defined for aseptic synovial fluid, aseptic female urine, and aseptic male urine, thus the ground truth may vary across material types.

Whole genome sequenced (reference) isolates from the LOD and septic urine cultures were processed as described previously [9]. Detected markers were used to determine the performance of the ARESdb AMR panel.

#### 2.3.3. Bioinformatics NGS Data Analysis

The number of trimmed and deduplicated reads, percentage of on-target reads, enrichment factor, average depth, and reads per kilobase million (RPKM) distributions of true positive (TP) and false positive (FP) markers were used for comparison. Bowtie2 v2.3.5.1 with a mapping quality of 20 (Fast gapped-read alignment with Bowtie 2) and samtools v1.7 (The Sequence Alignment/Map format and SAMtools) were used to calculate the number of reads on target. The enrichment factor was calculated as the percentage of reads on target from the enriched sample divided by the percentage of reads on target of the metagenomics sequenced sample. The average depth of each marker was calculated using bowtie2 by local alignment followed by Genomecov, bedtools v2.29.0 [22]. The RPKM was calculated by counting reads per target sequence with htseq-count v 0.13.5 [23].

$$RPKM=\frac{\#reads\;on\;gene\times 1\times 10^{-9}\;}{gene\;length\times \#total\;reads}$$

For marker comparison, markers detected by whole genome sequencing (WGS) were considered the ground truth. A true positive (TP) marker was defined as a marker detected both in the reference and in the sample; a false negative (FN) marker as marker detected in the reference and not detected in the sample; and a false positive (FP) marker as marker detected in the sample being analyzed and not present in the reference.

#### 2.3.4. Aseptic Sample Background Marker Removal

AMR markers chromosomally present in genera detected by 16S sequencing of blank aseptic samples were removed. Removed markers were not considered for comparison. Because marker removal was performed for each type of aseptic material independently, the resulting number of reference markers varied for the same species.

## 3. Results

We tested the performance of target-enriched sequencing with the ARESdb AMR panel versus metagenomics sequencing in different aseptic body fluid samples spiked with four different reference strains at concentrations of 10, 100, 1000, 10,000, and 100,000 CFU/mL.

### 3.1. ARESdb AMR Panel Sensitivity and LOD for Aseptic Synovial Fluids

Metagenomics sequencing of aseptic synovial fluid samples at an average read count per sample of 19,245,414 ± 3,903,155 reads proved insensitive, not detecting any AMR markers, across the range of spike-in concentrations. In contrast, AMR target-enriched samples achieved sensitivities of 91% ± 6% across all spiked-in reference strains at a concentration of 1000 CFU/mL with 3,679,296 ± 846,251 reads per sample. The *E. faecium* spike-in revealed a sensitivity of 86% already at 100 CFU/mL (Table 2).

The fraction of on-target reads increased with the spiked-in amounts of DNA from 6.4% (at 10 CFU/mL) to 40.8% (at 100,000 CFU/mL) across all spiked-in species (Table S1).

### 3.2. ARESdb AMR Panel Sensitivity and LOD in Aseptic Urine

#### 3.2.1. Male Urine

Metagenomics sequencing of aseptic male urine samples (Table 2) achieved an average sensitivity of 93% ± 7% for AMR marker detection across all species at a concentration of 100,000 CFU/mL. AMR target enrichment sequencing outperformed metagenomics sequencing, achieving comparable or better sensitivities of on average 97% ± 2% at already 10,000 CFU/mL for all reference strains. For two out of four reference strains, the sensitivity of target enrichment remained high down to 100 CFU/mL (91%, *E. faecium*) and 1000 CFU/mL (100%, *S. aureus*).

The percentage of reads on target for metagenomics sequencing increased across all species and concentrations from 0.004% to 0.225% compared with AMR target-enriched samples from 11.4% to 41.2% (Table S2). Of note, the percentage of reads on target obtained after enrichments depends on the enrichment and on the initial concentration of AMR marker target DNA. At 100,000 CFU/mL, true positive AMR markers were covered 99% ± 4% by AMR target-enriched sequencing and 96% ± 9% by metagenomics sequencing. For AMR target enrichment, the distribution of TP AMR marker depth (RPKM) was different between species, e.g., 2706 ± 1614 for *E. coli* and 26,046 ± 18,372 for *E. faecium* at 100,000 CFU/mL. For metagenomic sequencing, the distribution of TP AMR marker depth was on average 8 ± 4 for *E. coli* and 339 ± 419 for *E. faecium* at 100,000 CFU/mL (Figure S1).

#### 3.2.2. Female Urine

Metagenomics sequencing of aseptic female urine samples (Table 2) revealed a sensitivity of 93% only for the *E. faecium* sample at the highest concentration of 100,000 CFU/mL. In all other samples, only single (on average, sensitivity of 7 ± 22%) or no AMR markers were detected by metagenomics sequencing. In contrast, AMR target-enriched sequencing resulted in a sensitivity of on average 97 ± 3% at 1000 CFU/mL among all tested species. For *E. faecium*, the sensitivity of AMR target enrichment remained high (93%) down to 100 CFU/mL.

The percentage of reads on target for metagenomics sequencing increased across all species and concentrations from 0.015% to 0.034% compared with AMR target-enriched samples from 8.4% to 41.0% (Table S3). At 100,000 CFU/mL, true positive AMR markers were covered 99 ± 3% by AMR target-enriched sequencing and 93 ± 11% by metagenomics sequencing. For AMR target enrichment, the distribution of TP AMR marker depth (RPKM) was different between species, e.g., 2472 ± 1526 for *E. coli* and 26,694 ± 15,585 for *E. faecium* at 100,000 CFU/mL. For metagenomic sequencing, the distribution of TP AMR marker depth was on average 2 ± 1 for *E. coli* and 56 ± 54 for *E. faecium* at 100,000 CFU/mL samples (Figure S2).

### 3.3. Utility in Septic Urine Samples

For an initial proof-of-concept, sensitivity of the ARESdb AMR panel was tested against metagenomics sequencing and compared with whole genome sequenced isolates extracted from routine urine culture. The set of 13 septic urine samples spanned a total of six different species. Sample processing revealed high sensitivity of on average 99 ± 1% for both sequencing approaches. AMR target enrichment outperformed metagenomics sequencing by achieving on average 61% versus 1.5% on-target reads (Table 1 and Table 3). The coverage of TP AMR targeted markers by capture-based sequencing and metagenomics sequencing was on average 99 ± 4%. The distribution of target TP marker depth (RPKM) showed differences across septic samples with an average of 3162 ± 3355 for AMR target enrichment, while for metagenomics, the average depth obtained was 85 ± 35 (Figure S3).

State-of-the-art 16S rRNA sequencing directly from septic urine achieved 100% sensitivity among all patient samples. For all except one processed duplicate, the top genus detected was in concordance with the initial positive culture result. Duplicate 1 of patient ID-5 revealed the correct top genus (*Enterobacter* spp.), while duplicate 2 revealed *Enterobacter* spp. as third and *Klebsiella* spp. as most abundant genus.

## 4. Discussion

Rapid pathogen identification combined with AMR marker profiling directly from native samples is critical to unburden infectious disease management, antibiotic stewardship, and monitor trends in antibiotic resistance [24]. Despite the fact that current de-facto standard methods like PCR are highly sensitive, their target space is limited [8]. Previous studies have examined the utility of metagenomics sequencing in native sample types to overcome the limitations of bacterial culture [25,26,27,28,29]. Studies focusing on metagenomics sequencing of prosthetic joint infections have shown that the high proportion of human DNA background (>90%) represents a challenge for taxonomic identification, impeding sensitive antimicrobial resistance profiling [30,31,32]. Deep metagenomics sequencing has been shown to partially overcome host background and to sensitively recover microbial pathogens. However, compared with pathogen identification, considerably increased genome coverage is required to sensitively and reproducibly detect sparse genetic determinants of AMR. Therefore, routine use is hampered by sequencing depth and the associated sequencing costs [26].

Target capture-based methods have thus been proposed as a potentially viable approach for the identification of resistomes in complex native specimens [5,33]. We have selected over 9200 targets from the AMR biomarker database ARESdb and developed a target enrichment panel and NGS workflow, suitable for the processing and sensitive AMR profiling of complex sample types.

Our primary objective was to compare the sensitivity of target enrichment-based AMR marker detection to metagenomics sequencing in synovial fluid and urine samples. In the present study, the ARESdb AMR panel achieved sensitivities exceeding 91% ± 6% at ≥1000 CFU/mL for AMR marker detection across all tested species for spiked-in synovial fluid samples. In addition, we tested the ARESdb AMR panel among healthy male and female urine samples separately to investigate gender-specific background differences of the urine microbiota [34]. Among both male and female spiked-in urine samples, targeted sequencing outperformed metagenomics sequencing by achieving sensitivities exceeding 90% already at 1000 CFU/mL for all species in female and for *E. faecium* and *S. aureus* in male urine spiked-in samples. The LOD of the ARESdb AMR panel is sufficient for the analysis of complicated UTI (classified at pathogen loads >100,000 CFU/mL) and other UTI classifications including catheter associated UTIs, which are associated with lower pathogen loads of ≥1000 CFU/mL [35,36].

Moreover, we demonstrated the clinical relevance of the ARESdb AMR panel by a proof-of-concept study on a set of septic urine samples. Characterized clinical samples with confirmed monoinfection and available culture results were processed to investigate the sensitivity of the AMR panel. Clinical data showed that the bacterial load in septic samples exceeded 10,000 CFU/mL. As a consequence, high sensitivity was achieved by AMR target enrichment sequencing as well as by deep metagenomics sequencing. Previous studies [27,29] suggested that metagenomics sequencing of samples originating from UTI is feasible for taxonomic identification and AMR profiling. However, according to the data presented here, the number of reads covering AMR markers was significantly higher for target enrichment sequencing compared with metagenomics sequencing (61% versus 1.5%) at comparable numbers of total reads per sample (15,287,091 ± 6250 versus 18,997,857 ± 4,996,755). This indicates that the ARESdb AMR panel is a suitable application for lower sequencing depths. In addition, to complement the established AMR target enrichment workflow with taxonomic identification, we applied state-of-the art 16S rRNA sequencing, which was used to confirm the initial positive culture results (100% sensitivity). Of note, the current workflow is limited by its ability to link AMR markers with individual species in a multi-infection sample. This important limitation is neither addressed by molecular methods such as metagenomics sequencing or PCR nor the capture panel presented herein and will require further technological improvements.

## 5. Conclusions

The newly developed ARESdb AMR panel together with the established data analysis pipeline allowed to sensitively screen for >9200 AMR markers from samples with low concentrations of microbial DNA relative to human host DNA. For LOD experiments, AMR target enrichment achieved sensitivities >90% across all spiked-in reference strains at a concentration of 1000 CFU/mL. Our method was shown to be superior to metagenomics sequencing in the LOD experiments performed, sensitively capturing the resistome at lower sequencing capacity. In addition, culture-free AMR marker detection and taxonomic identification was demonstrated on a set of septic urine samples with high sensitivity (99%). Summarizing, our findings indicate the ARESdb AMR panel can be a valuable tool for comprehensive and sensitive AMR marker detection directly from body fluids.

## Figures and Tables

**Table 1:** Set of septic urine samples.

Sample ID	Gender	Sample Type	Type of UTI	Presence/Absence of Leukocytes (+/−)	Culture Pos. Detected Species	Antibiotic Treatment	CFU/mL
ID-1	m	MSU	recurrent	−	*E. coli*	Fosfomycin	>10,000
ID-2	m	MSU	recurrent	+	*E. coli ESBL*	none	>10,000
ID-3	m	MSU	recurrent	+	*E. coli*	none	>10,000
ID-4	m	MSU	recurrent	+	*C. koseri*	none	>10,000
ID-5	m	CU	acute	−	*E. hormaechei*	none	>10,000
ID-6	m	MSU	recurrent	−	*E. coli ESBL*	none	>10,000
ID-7	f	MSU	recurrent	+	*Enterococcus sp.*	none	>10,000
ID-8	f	MSU	acute	−	*E. coli*	none	>10,000
ID-9	f	MSU	acute	−	*K. pneumoniae*	none	>10,000
ID-10	f	MSU	acute	+	*E. coli*	none	>10,000
ID-11	f	MSU	acute	−	*Enterococcus sp.*	none	>10,000
ID-12	m	CU	acute	+	*E. coli ESBL*	none	>10,000
ID-13	f	CU	acute	+	*E. coli*	none	>10,000

MSU: midstream urine; CU: catheter urine; ESBL: extended spectrum-beta-lactamase; CFU: colony forming units; f: female; m: male.

**Table 2:** Sensitivity of the ARESdb AMR panel versus metagenomics sequencing for spiked-in native materials.

		Sensitivity Synovial Fluid		Sensitivity Male Urine		Sensitivity Female Urine	
Species	[CFU/mL]	MG	ARESdb AMR Panel	MG	ARESdb AMR Panel	MG	ARESdb AMR Panel
*K. quasipneumoniae*	10	0%/0%	18%/5%	0%/0%	0%/0%	0%/0%	0%/4%
	100		53%/42%		4%/4%		44%/12%
	1000		97%/95%		40%/66%		96%/94%
	10,000		97%/97%	8%/10%	98%/98%		98%/98%
	100,000		97%/97%	90%/98%	98%/96%	4%/6%	98%/98%
*E. faecium*	10	0%/0%	33%/22%	0%/0%	6%/63%	0%/0%	67%/73%
	100		83%/89%		88%/94%		93%/93%
	1000		89%/89%	25%/31%	94%/94%		93%/93%
	10,000		94%/94%	88%/94%	94%/94%	33%/53%	93%/93%
	100,000		94%/94%	94%/94%	94%/94%	93%/93%	93%/93%
*S. aureus*	10	0%/0%	0%/0%	0%/0%	0%/0%	0%/0%	0%/0%
	100		21%/7%				8%/8%
	1000		86%/79%		100%/100%		100%/100%
	10,000		100%/100%		100%/100%		100%/100%
	100,000		100%/100%	100%/100%	100%/100%		100%/100%
*E. coli*	10	0%/0%	36%/45%	0%/0%	0%/1%	0%/0%	0%/0%
	100		73%/77%		0%/1%		36%/41%
	1000		95%/95%		58%/57%		97%/99%
	10,000		95%/95%	1%/−	99%/97%		97%/97%
	100,000		95%/95%	78%/88%	97%/97%	1%/14%	99%/99%

Comparison of sensitivity for metagenomics sequencing (MG) and target-enriched sequencing (ARESdb AMR panel). Sensitivity is represented for performed duplicates (Duplicate 1/Duplicate 2).

**Table 3:** Performance overview for septic urine samples with confirmed monoinfection.

		Markers			Sensitivity		Reads on Target		Enrichment Factor
Sample ID	Species	WGS Isolate	MG	ARESdb AMR Panel	MG	ARESdb AMR Panel	MG	ARESdb AMR Panel	ARESdb AMR Panel
ID-1	*E. coli*	85	84/84	84/84	99%/99%	99%/99%	1.44%/1.45%	67%/68%	47/47
ID-2	*E. coli*	107	106/106	106/106	99%/99%	99%/99%	2.32%/2.67%	60%/60%	26/23
ID-3	*E. coli*	104	102/103	103/102	98%/99%	99%/98%	1.50%/1.45%	65%/65%	43/45
ID-4	*C. koseri*	45	43/43	43/42	96%/96%	96%/93%	0.82%/0.80%	34%/35%	41/44
ID-5	*E. hormaechei*	47	46/46	46/46	98%/98%	98%/98%	1.38%/1.38%	62%/62%	45/45
ID-6	*E. coli*	119	118/118	118/118	99%/99%	99%/99%	2.30%/2.29%	60%/61%	26/27
ID-7	*E. faecalis*	8	8/8	8/8	100%/100%	100%/100%	0.80%/0.62%	73%/73%	92/118
ID-8	*E. coli*	89	87/87	87/87	98%/98%	98%/98%	2.59%/2.64%	69%/69%	26/26
ID-9	*K. pneumoniae*	51	50/50	50/50	98%/98%	98%/98%	1.14%/1.11%	52%/52%	45/47
ID-10	*E. coli*	91	90/90	90/90	99%/99%	99%/99%	1.57%/1.48%	61%/60%	39/41
ID-11	*Enterococcus* sp.	9	9/9	9/9	100%/100%	100%/100%	0.71%/0.72%	69%/70%	97/97
ID-12	*E. coli*	109	108/108	108/107	99%/99%	99%/98%	1.48%/1.48%	58%/58%	39/39
ID-13	*E. coli*	101	100/100	100/100	99%/99%	99%/99%	1.96%/1.86%	67%/67%	34/36
AVERAGE		74	73	73	99%	99%	1.54%	61%	47

Comparison of performance parameters for metagenomics sequencing (MG) and target-enriched sequencing (ARESdb AMR panel). The number of ground truth markers was defined based on the whole genome sequence of the bacterial isolate (WGS isolate). Samples were processed as duplicates and represented as (Duplicate 1/Duplicate 2).

## Data Availability

NGS datasets generated for this study can be found in the DDBJ/EMBL/GenBank; accession PRJNA743171.

## Supplementary Materials

The following are available online at https://www.mdpi.com/article/10.3390/microorganisms9081672/s1, Figure S1: Marker coverage and depth of metagenomics and target enriched male urine samples; Figure S2: Marker coverage and depth of metagenomics and target enriched female urine samples; Figure S3: Marker coverage and depth of metagenomics and target enriched septic urine samples; Table S1: Performance overview for aseptic synovial fluid samples spiked with four different pathogens; Table S2. Performance overview for aseptic male urine samples spiked with four different pathogens; Table S3. Performance overview for aseptic female urine samples spiked with four different pathogens.
