# Supplementary material for: Culture-Free Detection of Antibiotic Resistance Markers from Native Patient Samples by Hybridization Capture Sequencing
Source: Microorganisms. 2021 Aug 5;9(8):1672. doi: 10.3390/microorganisms9081672 (PMC8398375; doi:10.3390/microorganisms9081672)
Supplement: Supplementary file 1 [file microorganisms-09-01672-s001.zip › MS Table S1-3_20210701.pdf]

Table S1. Performance overview for aseptic synovial fluid samples spiked with four different pathogens.

		**Markers**			**Sensitivity [%]**		**reads on target [%]**		**Enrichment Factor**
**Species**	**[CFU/ml]**	WGS Isolate	MG	ARESdb AMR panel	MG	ARESdb AMR panel	MG	ARESdb AMR panel	ARESdb AMR panel
K. quasipneumoniae	**10**	38	0/0	7/2	0%/0%	18%/5%	0.0035%/0.0004%	7%/6%	2,006/13,141
	**100**			20/16		53%/42%	0.0004%/0.0051%	6%/3%	12,464/653
	**1 000**			37/36		**97%/95%**	0.0186%/0.0060%	9%/9%	495/1,442
	**10 000**			37/37		**97%/97%**	0.0441%/0.0062%	20%/22%	452/3,547
	**100 000**			37/37		**97%/97%**	0.0048%/0.0053%	36%/36%	7,501/6,847
E. faecium	**10**	18	0/0	6/4	0%/0%	33%/22%	0.0099%/0.0019%	5%/12%	495/6,313
	**100**			15/16		**83%/89%**	0.0028%/0.0001%	5%/4%	1,836/40,261
	**1 000**			16/16		**89%/89%**	0.0017%/0.0001%	7%/8%	4,147/88,072
	**10 000**			17/17		**94%/94%**	0.0002%/0.0054%	16%/17%	65,914/3,161
	**100 000**			17/17		**94%/94%**	0.0042%/0.0015%	36%/36%	8,604/24,883
S. aureus	**10**	14	0/0	0/0	0%/0%	0%/0%	0.1085%/0.0060%	6%/5%	54/884
	**100**			3/1		21%/7%	0.0069%/0.1088%	45%/5%	6,573/44
	**1 000**			12/11		**86%/79%**	0.0079%/0.1367%	15%/6%	1,927/42
	**10 000**			14/14		**100%/100%**	0.0041%/0.0078%	11%/10%	2,619/1,335
	**100 000**			14/14		**100%/100%**	0.0018%/0.1065%	33%/34%	17,913/323
E. coli	**10**	22	0/0	8/10	0%/0%	36%/45%	0.0028%/0.0062%	5%/5%	1,844/781
	**100**			16/17		73%/77%	0.0034%/0.2153%	16%/6%	4,724/30
	**1 000**			21/21		**95%/95%**	0.0846%/0.0031%	14%/14%	171/4,697
	**10 000**			21/21		**95%/95%**	0.0061%/0.0089%	37%/42%	6,003/4,696
	**100 000**			21/21		**95%/95%**	0.0077%/0.0100%	58%/57%	7,487/5,732

Comparison of performance parameters for metagenomics sequencing (MG) and targeted enriched sequencing (ARESdb AMR panel). The number of ground truth markers was defined based on the whole genome sequence of the bacterial isolate (WGS Isolate). Samples were processed as duplicates and are represented as [Duplicate 1/ Duplicate 2].

Table S2. Performance overview for aseptic male urine samples spiked with four different pathogens.

		Markers			Sensitivity [%]		reads on target [%]		Enrichment Factor
**Species**	**[CFU/ml]**	WGS Isolate	MG	ARESdb AMR panel	MG	ARESdb AMR panel	MG	ARESdb AMR panel	ARESdb AMR panel
K. quasipneumoniae	10	50	0/0	0/0	-/-	0%/0%	0.0009%/0.0018%	8%/13%	9,082/7,246
	100			2/2		4%/4%	0.0014%/0.0010%	9%/10%	6,113/9,669
	1 000			20/33		**40%/66%**	0.0109%/0.0030%	27%/28%	2,471/9,438
	10 000		4/5	49/49	8%/10%	**98%/98%**	0.0164%/0.0373%	33%/33%	2,015/891
	100 000		45/49	49/48	**90%/98%**	**98%/96%**	0.1167%/0.1548%	35%/33%	303/216
E. faecium	10	16	0/0	1/10	-/-	6%/63%	0.0020%/0.0021%	15%/15%	7,400/7,349
	100			14/15		**88%/94%**	0.0027%/0.0025%	11%/13%	4,006/5,232
	1 000		4/5	15/15	25%/31%	**94%/94%**	0.0352%/0.0052%	28%/28%	790/5,360
	10 000		14/15	15/15	**88%/94%**	**94%/94%**	0.0490%/0.0563%	29%/30%	601/530
	100 000		15/15	15/15	**94%/94%**	**94%/94%**	0.4327%/0.5358%	34%/34%	78/64
S. aureus	10	3	0/0	0/0	-/-	0%/0%	0.0016%/0.0013%	9%/11%	6,028/8,599
	100						0.0018%/0.0018%	5%/7%	2,653/4,020
	1 000			3/3		**100%/100%**	0.0023%/0.0144%	26%/28%	11,595/1,960
	10 000			3/3		**100%/100%**	0.0086%/0.0100%	36%/36%	4,123/3,604
	100 000		3/3	3/3	**100%/100%**	**100%/100%**	0.1089%/0.1242%	43%/42%	397/342
E. coli	10	77	0/0	0/1	-/-	0%/1%	0.0108%/0.0023%	9%/11%	797/4,761
	100			0/1		0%/1%	0.0022%/0.0289%	9%/9%	4,107/302
	1 000			45/44		**58%/57%**	0.0043%/0.0043%	43%/39%	9,974/11,360
	10 000		1/0	76/75	1%/-	**99%/97%**	0.0187%/0.0243%	53%/52%	2,856/2,154
	100 000		60/68	75/75	**78%/88%**	**97%/97%**	0.1414%/0.1824%	54%/54%	384/297

Comparison of performance parameters for metagenomics sequencing (MG) and targeted enriched sequencing (ARESdb AMR panel). The number of ground truth markers was defined based on the whole genome sequence of the bacterial isolate (WGS Isolate). Samples were processed as duplicates and are represented as [Duplicate 1/ Duplicate 2].

Table S3. Performance overview for aseptic female urine samples spiked with four different pathogens.

		Markers			Sensitivity [%]		reads on target [%]		Enrichment Factor
**Species**	**[CFU/ml]**	WGS Isolate	MG	ARESdb AMR panel	MG	ARESdb AMR panel	MG	ARESdb AMR panel	ARESdb AMR panel
K. quasipneumoniae	10	52	0/0	0/2	0%/0%	0%/4%	0.0032%/0.0067%	9%/8%	2,797/1,201
	100			23/6		44%/12%	0.0147%/0.0023%	7%/7%	484/2,983
	1 000			50/49		**96%/94%**	0.0034%/0.0037%	11%/10%	3,331/2,801
	10 000			51/51		**98%/98%**	0.0059%/0.0100%	21%/24%	3,562/2,363
	100 000		2/3	51/51	4%/6%	**98%/98%**	0.0136%/0.0150%	32%/32%	2,348/2,149
E. faecium	10	15	0/0	10/11	0%/0%	67%/73%	0.0309%/0.0070%	8%/8%	269/1,154
	100			14/14		**93%/93%**	0.0173%/0.0032%	9%/8%	507/2,478
	1 000			14/14		**93%/93%**	0.0024%/0.0039%	14%/13%	5,615/3,313
	10 000		5/8	14/14	33%/53%	**93%/93%**	0.0077%/0.0080%	31%/31%	4,054/4,018
	100 000		14/14	14/14	**93%/93%**	**93%/93%**	0.0816%/0.0890%	39%/39%	475/434
S. aureus	10	13	0/0	0/0	0%/0%	0%/0%	0.0027%/0.0031%	8%/8%	3,014/2,750
	100			1/1		8%/8%	0.0039%/0.0042%	8%/8%	2,025/1,857
	1 000			13/13		**100%/100%**	0.0032%/0.0023%	9%/9%	2,794/3,900
	10 000			13/13		**100%/100%**	0.0170%/0.0042%	19%/18%	1,113/4,317
	100 000			13/13		**100%/100%**	0.0082%/0.0106%	37%/38%	4,526/3,593
E. coli	10	76	0/0	0/0	0%/0%	0%/0%	0.0632%/0.0036%	10%/8%	151/2,169
	100			27/31		36%/41%	0.0041%/0.0326%	14%/8%	3,518/257
	1 000			74/75		**97%/99%**	0.0024%/0.0052%	14%/155	5,910/2,903
	10 000			74/74		**97%/97%**	0.0056%/0.0051%	38%/40%	6,839/7,753
	100 000		1/11	75/75	1%/14%	**99%/99%**	0.0230%/0.0312%	55%/56%	2,374/1,789

Comparison of performance parameters for metagenomics sequencing (MG) and targeted enriched sequencing (ARESdb AMR panel). The number of ground truth markers was defined based on the whole genome sequence of the bacterial isolate (WGS Isolate). Samples were processed as duplicates and are represented as [Duplicate 1/ Duplicate 2].
