# Supplementary figures and images for: Culture-Free Detection of Antibiotic Resistance Markers from Native Patient Samples by Hybridization Capture Sequencing
Source: Microorganisms. 2021 Aug 5;9(8):1672. doi: 10.3390/microorganisms9081672 (PMC8398375; doi:10.3390/microorganisms9081672)

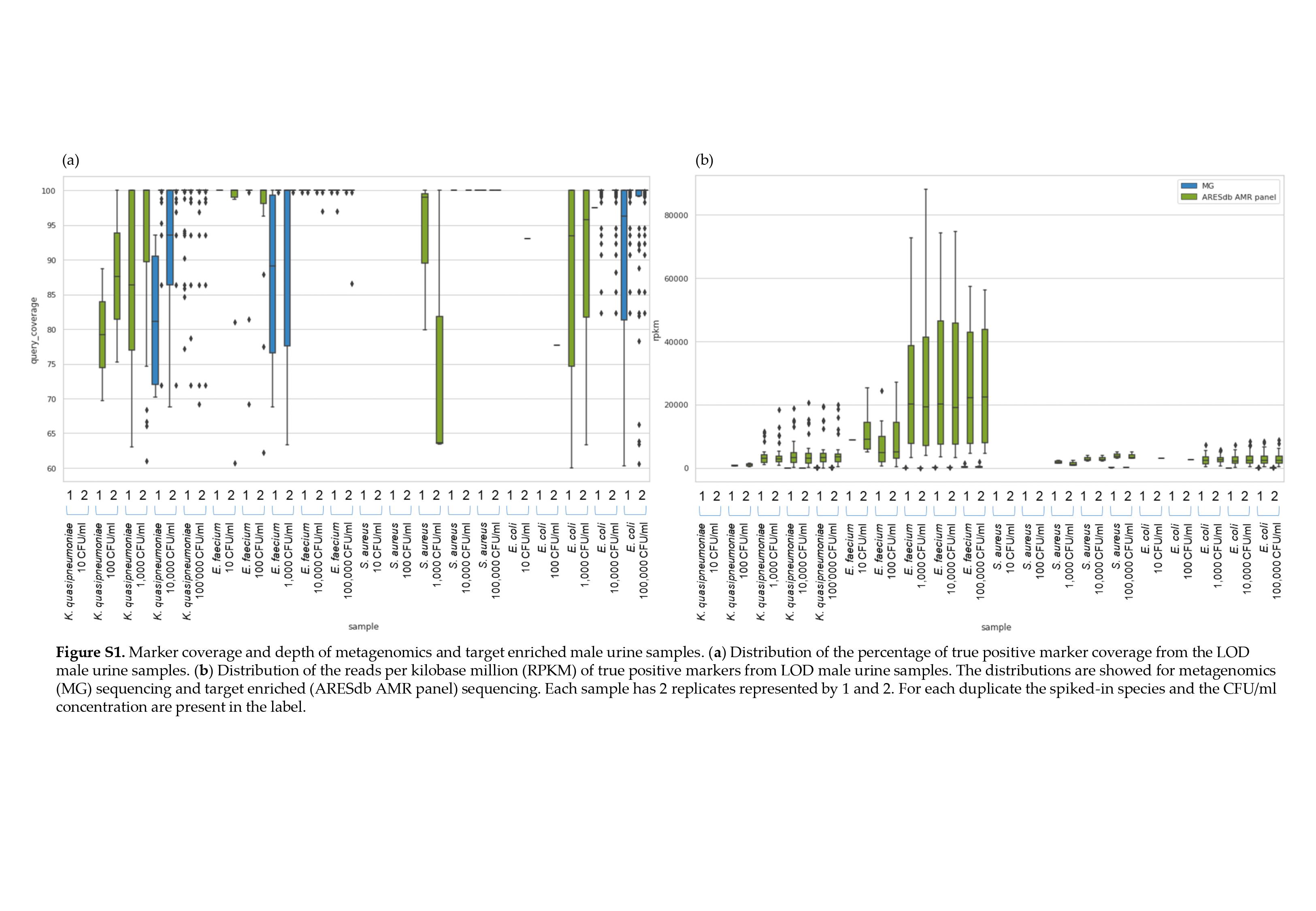

Supplement: Supplementary file 1 [file microorganisms-09-01672-s001.zip › MS figure S1.jpg]

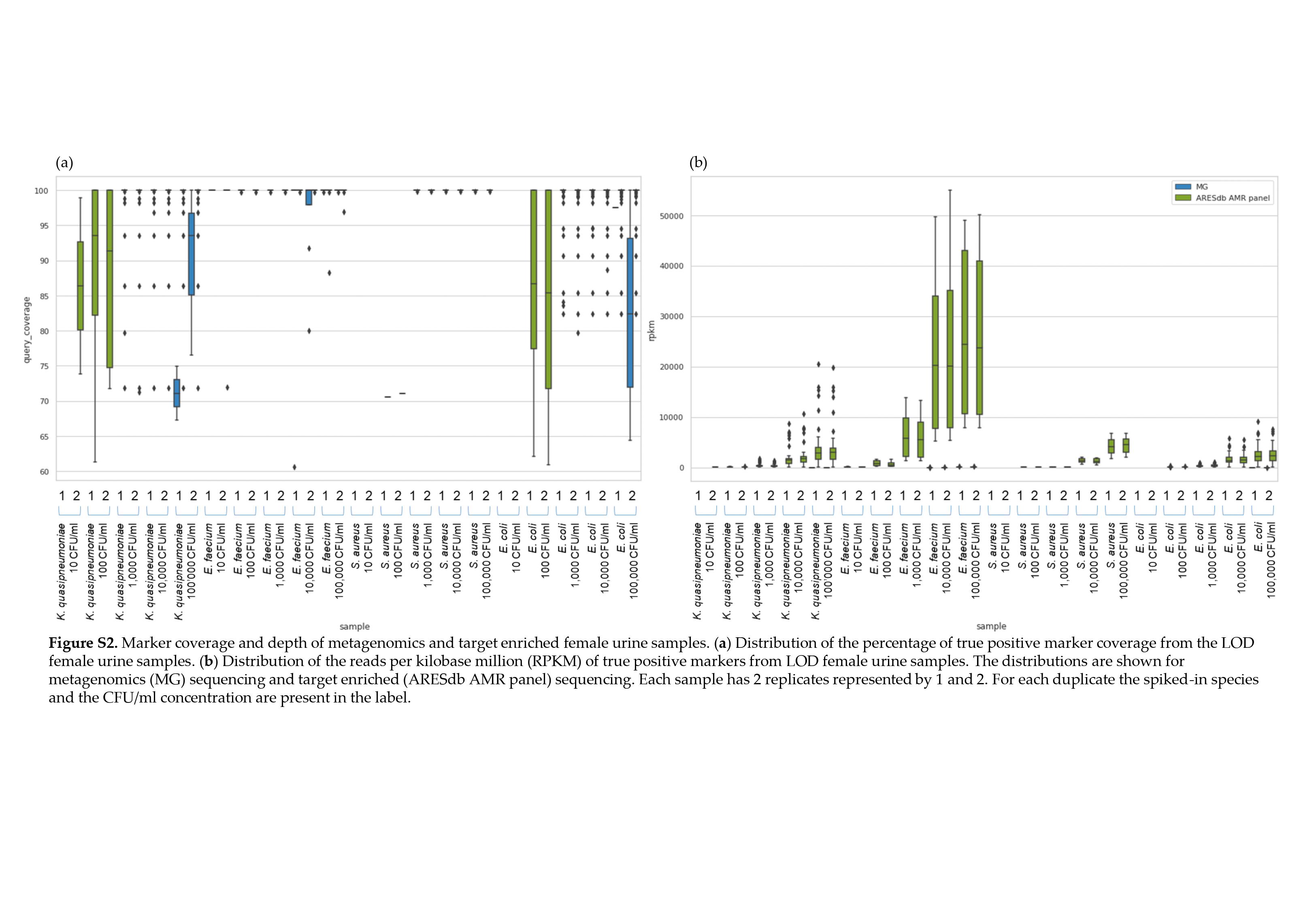

Supplement: Supplementary file 1 [file microorganisms-09-01672-s001.zip › MS figure S2.jpg]

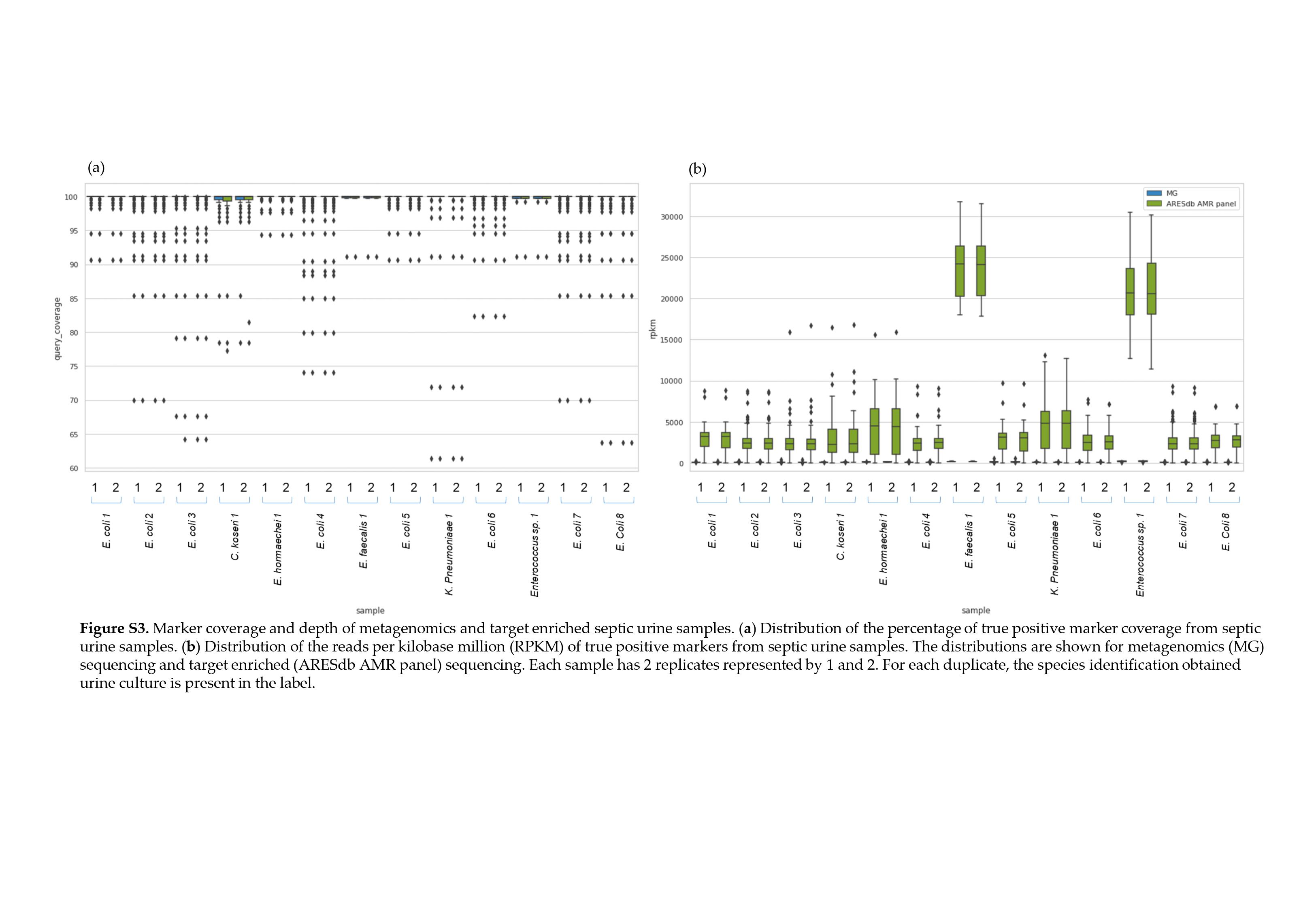

Supplement: Supplementary file 1 [file microorganisms-09-01672-s001.zip › MS figure S3.jpg]
